# Evaluation of performance for IBARAKI biological crystal diffractometer iBIX with new detectors

**DOI:** 10.1107/S0909049513021845

**Published:** 2013-10-11

**Authors:** Katsuhiro Kusaka, Takaaki Hosoya, Taro Yamada, Katsuaki Tomoyori, Takashi Ohhara, Masaki Katagiri, Kazuo Kurihara, Ichiro Tanaka, Nobuo Niimura

**Affiliations:** a Frontier Research Center for Applied Atomic Sciences, Ibaraki University, 162-1 Shirakata, Tokai, Ibaraki 319-1106, Japan; b Center for Neutron Science and Technology, Comprehensive Research Organization for Science and Society, 161-1 Shirakata, Tokai, Ibaraki 319-1106, Japan; c J-PARC Center, Japan Atomic Energy Agency, 2-4 Shirane Shirakata, Tokai, Ibaraki 319-1195, Japan; d Quantum Beam Science Directorate, Japan Atomic Energy Agency, 2-4 Shirane Shirakata, Tokai, Ibaraki 319-1195, Japan

**Keywords:** neutron protein crystallography, TOF single-crystal diffractometer

## Abstract

The IBARAKI biological crystal diffractometer, iBIX, is a high-performance time-of-flight neutron single-crystal diffractometer for elucidating mainly the hydrogen, protonation and hydration structures of biological macromolecules in various life processes. Since the end of 2008, iBIX has been available to users’ experiments supported by Ibaraki University. Since August 2012, an upgrade of the 14 existing detectors has begun and 16 new detectors have been installed for iBIX. The total measurement efficiency of the present diffractometer has been improved by one order of magnitude from the previous one with the increasing of accelerator power. In December 2012, commissioning of the new detectors was successful, and collection of the diffraction dataset of ribonucrease A as a standard protein was attempted in order to estimate the performance of the upgraded iBIX in comparison with previous results. The resolution of diffraction data, equivalence among intensities of symmetry-related reflections and reliability of the refined structure have been improved dramatically. iBIX is expected to be one of the highest-performance neutron single-crystal diffractometers for biological macromolecules in the world.

## Abstract 2

The time-of-flight neutron single-crystal diffractometer iBIX at the next-generation neutron source J-PARC has been upgraded and is available for user experiments on protein samples in particular. Neutron structure analysis of a standard protein sample was carried out in order to evaluate the performance of iBIX.

## 1. Introduction

Hydrogen atoms and water molecules around proteins play a very important role in the stability of the three-dimensional structure and in many physiological functions of them. Recently, the demand on positional information of hydrogen atoms in a protein molecule has been increasing after many projects in the world in which thousands of protein structures have been analyzed. Neutron diffraction can provide an experimental method of directly identifying hydrogen atoms in proteins as a technique complementary to ultra-high-resolution X-ray diffraction.

Recently, the development of a neutron imaging plate (NIP) and a diffractometer equipped with a NIP on reactor neutron sources has been a breakthrough event in neutron protein crystallography and has met the demand on structural biology in the life science field (Niimura & Bau, 2008 ▶). Although the demand on neutron structural biology in the current life science field has increased continuously, neutron protein crystallography (NPC) still remains to this day a severely limited technique. To meet the increasing demand on neutron structural biology, Ibaraki Prefectural Government in Japan has started to construct a new high-performance time-of-flight (TOF) neutron single-crystal diffractometer (IBARAKI Biological Crystal Diffractometer, iBIX) at the 1 MW pulsed neutron source in J-PARC, for industrial use since 2004. The diffractometer is designed to measure samples with their cell edges up to around 150 Å with a resolution up to 1.2 Å in the case of biological macromolecules, and a resolution up to 0.7 Å in the case of organic compounds. It should be possible to collect a full data set in three to four days for a crystal of volume 2 mm^3^ and/or in about one month for a crystal of volume 0.1 mm^3^ of a biological macromolecule. In this paper the current status of iBIX, and the upgraded diffractometer in particular, will be presented.

## 2. Basic design of iBIX before upgrading

The iBIX was funded by Ibaraki Prefecture. It was constructed by the iBIX Development Team which consists of members of Ibaraki University and JAEA. The iBIX bas been installed on beamline port No. 3 with a coupled moderator at the Material and Life Science Facility. The coupled moderator can provide a more intense peak and integrated intensity of neutron flux but a wider pulse shape than a decoupled one in order to obtain accurate integrated intensities of weak Bragg reflections around the resolution limit from a single-crystal sample of a protein.

The optics parameters (beam divergence and distance between sample and detectors: *L*_2_) have been determined by considering the overlapping of Bragg reflection spots using the original simulation program (Kusaka *et al.*, 2006 ▶). A super-mirror guide tube of the iBIX was designed based on the determined optics parameters (Tanaka *et al.*, 2009 ▶). A curved guide tube has been selected to protect sample proteins from high-energy γ-rays and neutrons without a T_0_ chopper, and the well designed tapered guide tube can serve to reduce the reflection number of neutrons on the surface of the super-mirror so that more neutrons arrive at the sample position.

To realise high efficiency, iBIX requires a completely new detector system. A two-dimensional position-sensitive detector using ZnS(Ag/^10^B_2_O_3_) scintillator with a wavelength shift fiber system which has a high spatial resolution, a smaller dead area, a high counting rate and high efficiency was developed (Hosoya *et al.*, 2009 ▶). Since the end of 2008, 16 detectors have been installed on iBIX. The total amount of the solid angle of the diffractometer subtended by the 14 detectors was 9.1% for 4π, and iBIX has been available to user experiments supported by Ibaraki University (Tanaka *et al.*, 2010 ▶).

A gas-flow-type cooling system as a sample environment is also available for low-temperature experiments. N_2_ gas can be generated from air by pressure swing adsorption equipment, while He gas should be supplied from a He gas cylinder.

## 3. Upgrading existing detectors and installing new detectors

**Figure 1:**
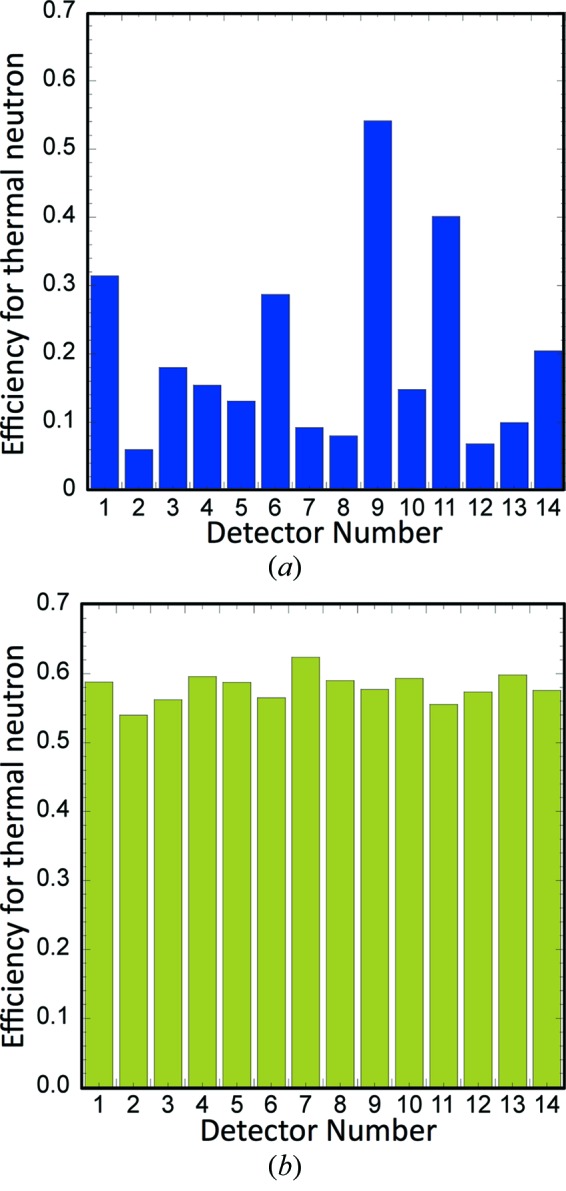
Efficiencies both before (*a*) and after (*b*) upgrading for each existing detector.

Since August 2012, the new detector system has been adapted such that the 14 existing detectors have been upgraded and 16 new detectors have been installed for iBIX. The new system has been improved dramatically in terms of the detector efficiency, uniformity of sensitivity, spatial resolution and maintenance. The upgraded parts of the 14 existing detectors are as follows: the scintillator packs including scintillators, the wavelength shift fibers strung for the *x* and *y* direction (256 × 256 pixels), the light guide units and the encoder modules. Fig. 1 ▶ shows the efficiencies both before (*a*) and after (*b*) upgrading for each existing detector. Average efficiencies of the 14 existing detectors both before and after upgrading are 19% and 58%, mainly due to installing the newly developed scintillators and improving the bending method of the wavelength shift fibers at the edge of the detector, respectively. The average efficiencies after upgrading become almost three times larger than before upgrading; therefore the measurement efficiency of the diffractometer for a full data set is expected to become almost three times higher than that before upgrading. Uniformities of detector-sensitivity in the direction of the *x* and *y* axes before upgrading was about 20% for detector No. 3. After upgrading, it was reduced to about 10%; therefore the accuracy of the integrated intensities for Bragg reflections is expected to improve.

**Figure 2:**
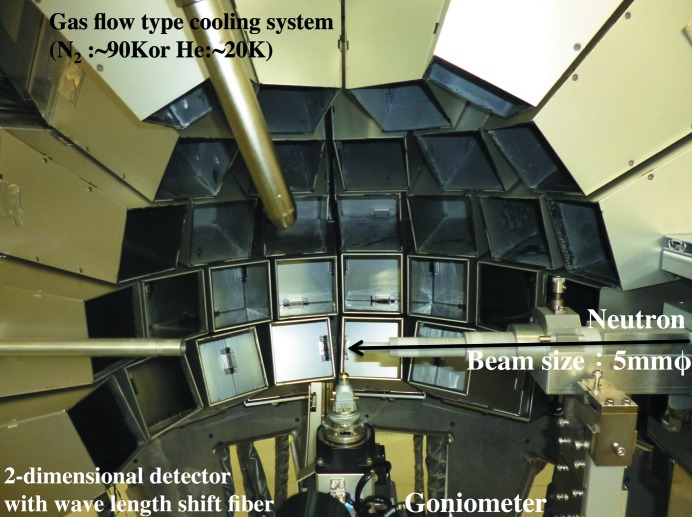
Inside the new diffractometer for iBIX.

At the end of 2012, accelerator power increased from 200 kW to 300 kW. The total measurement efficiency of the present iBIX has been improved by almost one order of magnitude from the previous one with the increasing of accelerator power. The final specifications of the new iBIX are shown in Table 1 ▶. In December 2012, the commissioning (installation of the diffractometer, noise reduction, determination of the measurement parameter for each detector, the test of the data acquisition by all detectors *etc*.) of the new detector system was successful. The new iBIX is shown in Fig. 2 ▶.

## 4. Diffraction measurement of standard samples and their structure refinement

**Figure 3:**
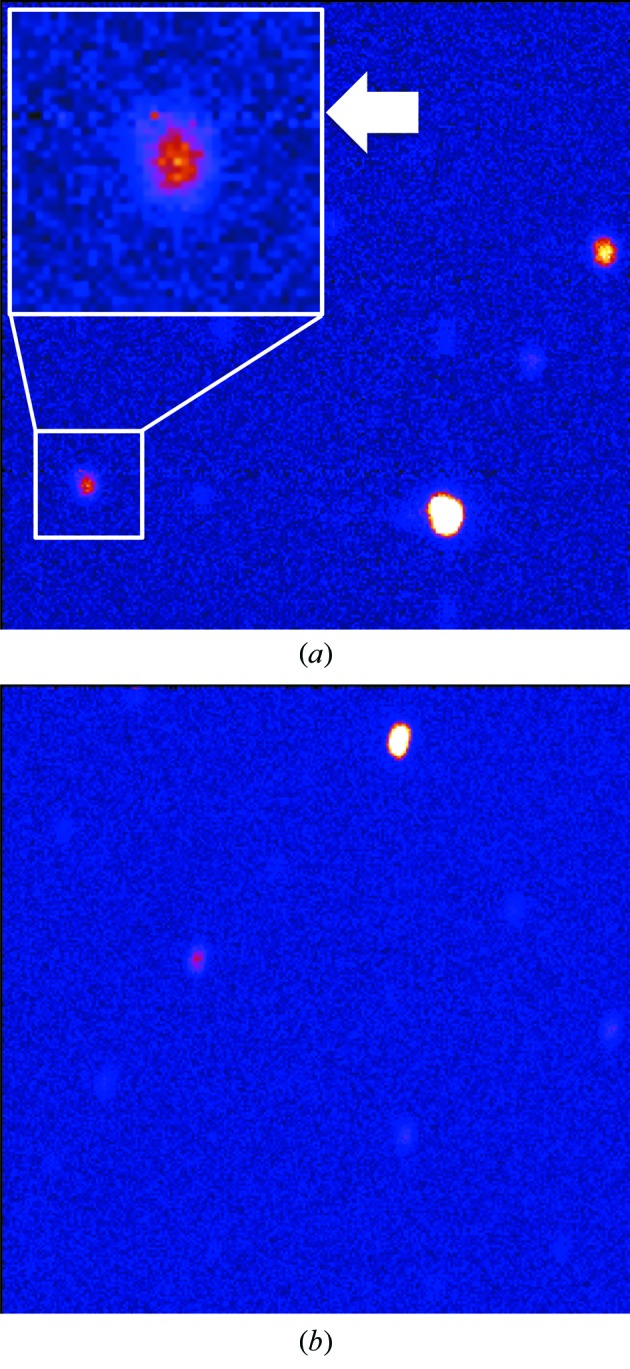
TOF diffraction pattern of ABT measured by using the detector before (*a*) and after (*b*) upgrading.

After measurement of the incoherent neutron scattering of a vanadium sphere (4.8 mm in diameter) as calibration data for the uniformity of sensitivity among pixels of the detector and the wavelength dependence of the incident neutron, a diffraction experiment of a single crystal of ammonium bitartrate (ABT) as a standard sample was carried out using the new iBIX. TOF diffraction patterns of ABT measured by using the detector system before and after upgrading are shown in Figs. 3(*a*) and 3(*b*) ▶, respectively. There are some low-counting pixels [one example is shown by a white arrow in Fig. 3(*a*) ▶] in the diffraction pattern using the detectors before upgrading because the sensitivity of this line is lower than that of the other lines and even calibrated data of the low-counting pixels could not reach sufficient accuracy, because the accuracy of the intensity of the Bragg reflection observed on the low-counting pixels should be reduced. However, there is no low-counting pixel in the diffraction pattern using the new detector system after upgrading and all Bragg reflections appear very clearly.

The TOF neutron diffraction dataset of ribonuclease A (RNase A) as a standard protein sample for neutron structure analysis has been collected using the new iBIX in order to estimate the performance and characteristics of the new iBIX after upgrading the diffractometer.

Bovine pancreatic ribonuclease A (type XII-A: R5500, Lot No. 055K7695) was purchased from Sigma-Aldrich. A 45% aqueous solution of *tert*-butyl alcohol was poured gently onto lyophilized RNase A. The final concentration of RNase A was 6.0 mg ml^−1^. The mixture was kept at 298 K. The crystal grew to 6.0 mm^3^ (2.5 mm × 2.4 mm × 1 mm) after two weeks. The crystal was soaked in deuterated solution for ten days to reduce background scattering from H atoms, which have a large incoherent neutron scattering length.

The TOF diffraction pattern was measured as a preliminary measurement in order to check the quality of the single-crystal sample, resolution and to determine the exposure time and crystal orientation for the measurement of a full data set. The number of crystal settings and each orientation were determined based on the crystal orientation obtained by pre­liminary measurement with the original computer program to calculate the ratio of the theoretically observable reflection number to the total number of reflections. Crystal data and measurement conditions are shown in Table 2 ▶.

**Figure 4:**
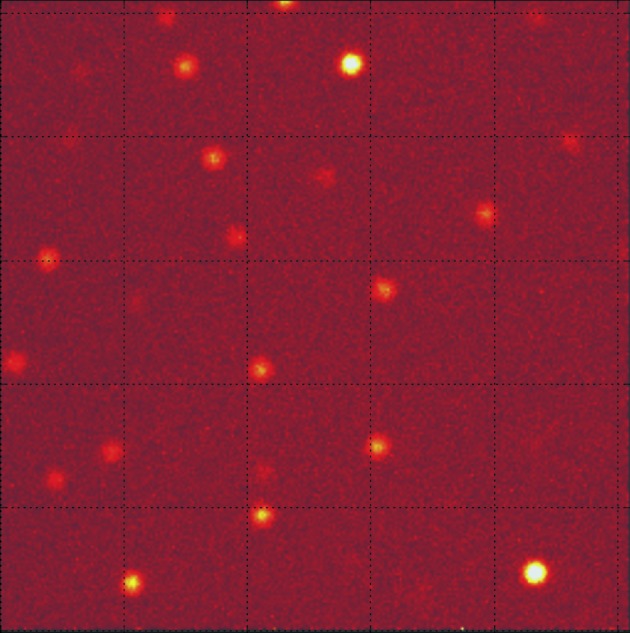
Example of TOF diffraction patterns of RNase A obtained by one detector located at 54° in 2θ.

The full data set for neutron structure analysis could be obtained in seven days at 280 kW accelerator power at J-PARC. The exposure time was 4 h per setting. The total number of settings was 40. An example of a TOF diffraction pattern of RNase A is shown in Fig. 4 ▶. Very sharp Bragg spots and a typical Laue diffraction pattern of a standard protein sample could be observed by using the new iBIX. Data reduction was carried out using the program *STARGazer* (Ohhara *et al.*, 2009 ▶) developed for TOF diffraction data based on *ISAW* (Argonne National Laboratory, USA). The data reduction was successfully completed and then the integrated intensities of the Bragg reflections could be obtained for the structure refinement. A total of 15820 unique reflections were obtained with an overall *R*_sym_ of 13.5% from 47166 observed reflections. A randomly selected 10% of reflections were assigned as a test set for cross validation. The structure refinement was carried out using the program *PHENIX-REFINE* including *PHENIX-1.8* (Adams *et al.*, 2010 ▶). The structure of RNase A solved with neutron diffraction data at 1.7 Å resolution (PDB code 3a1r; Yagi *et al.*, 2009 ▶) was used as the initial amplitude for the structure refinement. The final values of *R*_cryst_ and *R*_free_ were 18.1% and 23.6%, respectively, for 15820 unique reflections to a resolution of 1.5 Å. *R*_cryst_, *R*_free_ and the resolution of the data measured by using the detectors after upgrading were dramatically improved in comparison with those of the data measured by using the detectors before upgrading (*R*_cryst_: 20.7%; *R*_free_: 28.9%; resolution: 1.7 Å). The statistics of the data reduction and refinement are summarized in Table 3 ▶. Reasonable structure and data statistics could be obtained after the structure refinement by comparing with the already-reported structure. *R*_sym_ of the intensity data set of this study is higher than the reported one collected by BIX-3, JRR-3 (Yagi *et al.*, 2009 ▶). Major causes of this are assumed as follows: the intensities of Bragg reflections obtained from TOF diffraction data should be calibrated for the dependence of the intensity of the incident neutrons on wavelength; vanadium incoherent scattering as calibration data is not the perfect point source from the sample position. In the near future, the calibration method included in the data reduction software should be upgraded to improve the accuracy of the intensity data.

**Figure 5:**
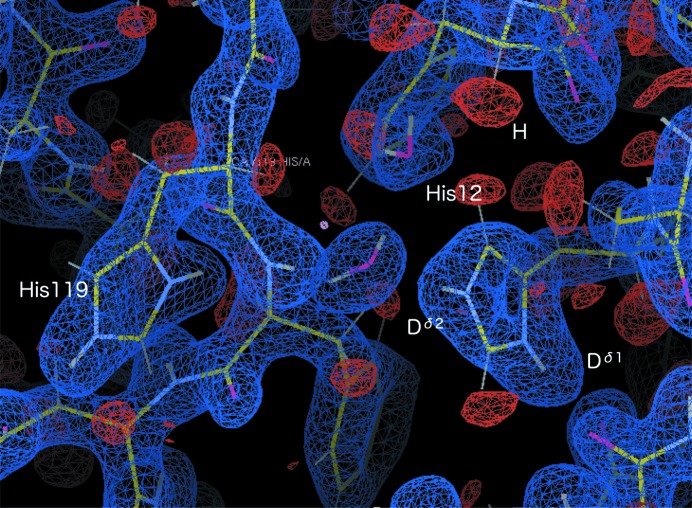
2|*F*_o_| − |*F*_c_| neutron-scattering-length map around the active site including His12 and His119. Blue: +1.5σ; red: −2.5σ.

The 2|*F*_o_| − |*F*_c_| neutron-scattering-length map around the active site including His12 and His119 is shown in Fig. 5 ▶ based on the final structure model of the refinement. It is important to know the protonation states of His12 and His119 in order to understand the hydrolysis mechanism of RNase A. The result of the neutron structure refinement of this study is the same as that of the structure already reported in recognizing the protonation states of them. The resolution of diffraction data, equivalence among intensities of symmetry-related reflections and reliability of the refined structure have been improved dramatically in comparison with those of the data of RNase A obtained by using iBIX before upgrading. iBIX is expected to be one of the highest performance neutron single-crystal diffractometers for biological macromolecules in the world.

## 5. Conclusion and future prospects

Upgrading of the 14 existing detectors and installing of 16 newly developed detectors were successfully completed; iBIX has been available for user experiments on protein samples in particular since January 2013.

In the future, the data reduction software will be upgraded in order to improve the accuracy of the data reduction for the integrated intensities of Bragg reflections and to become a more user-friendly system. For example, background extraction, data correction method, integration method and decentralized processing will be developed in the next phase. In the case of hardware, we will prepare the utility equipment for sample environment and the fast and friendly measurement system before the accelerator power becomes 1 MW.

## Figures and Tables

**Table 1:** Final specifications of the new iBIX

Pulse repetition (Hz)	25
Moderator	Coupled H_2_ (para) 100 mm ×100 mm
Guide tube	25
*L*_1_ (m)	40
*L*_2_ (m)	0.49
Maximum unit-cell length (Å)	∼150
Measurement region in *d* spacing (Å)	0.35 < *d* < 50
Range of neutron wavelength (Å)	0.5 *<* λ *<* 9.0 or more
Neutron flux (neutrons s^−1^ cm^−2^)	7 × 10^7^
Sample environment	Gas-flow-type cooling system, He: ∼20 K; N_2_: ∼90 K
Detector	Two-dimensional, scintillator, wavelength shift fiber type
Size of sensitive area (mm)	133 × 133
Spatial resolution (mm)	<1
Standard size of sample (mm^3^)	1
Standard measurement time (days)	0.5 for organic compounds, 3 for biological macromolecules

**Table 2:** Crystal data, measurement conditions and refinement statistics for ribonuclease A

Sample name		Ribonuclease A
Temperature (K)		293
Space group		*P*2_1_
Unit-cell	*a* (Å)	30.4
	*b* (Å)	38.6
	*c* (Å)	53.4
	β (°)	105.8
Crystal size (mm^3^)		6.0
Accelerator power (kW)		280
Range of wavelength (Å)		1.6–4.6
Exposure time (h)		4.0
Total number of settings		40
Total measurement time (days)		7

**Table 3:** Data reduction and refinement statistics

	iBIX	Already reported
No. of observed reflections	47166	31649
No. of unique reflections	15820	15039
Resolution (Å)	1.5 [*I* > 1.5σ(*I*)]	1.4
Completeness (%)	82.1	64.0
Average *I*/σ(*I*)	11.8	8.4
*R*_sym_ (%)	13.5	7.1
*R*_cryst_ (%)	18.1	19.5
*R*_free_ (%)	23.6	23.8
